# Acute and Subchronic Oral Toxicity Study of Polyherbal Formulation Containing *Allium sativum* L., *Terminalia bellirica* (Gaertn.) Roxb., *Curcuma aeruginosa* Roxb., and *Amomum compactum* Sol. ex. Maton in Rats

**DOI:** 10.1155/2020/8609364

**Published:** 2020-03-24

**Authors:** E. N. Sholikhah, M. Mustofa, D. A. A. Nugrahaningsih, F. S. Yuliani, S. Purwono, S. Sugiyono, S. Widyarini, N. Ngatidjan, J. Jumina, D. Santosa, M. Koketsu

**Affiliations:** ^1^Department of Pharmacology and Therapy, Faculty of Medicine Public Health and Nursing, Universitas Gadjah Mada, Yogyakarta 55281, Indonesia; ^2^Center of Herbal Medicine, Faculty of Medicine Public Health and Nursing, Universitas Gadjah Mada, Yogyakarta 55281, Indonesia; ^3^Department of Pathology, Faculty of Veterinary Medicine, Universitas Gadjah Mada, Yogyakarta 55281, Indonesia; ^4^Department of Chemistry, Faculty of Mathematics and Natural Sciences, Universitas Gadjah Mada, Yogyakarta 55281, Indonesia; ^5^Department of Pharmaceutical Biology, Faculty of Pharmacy, Universitas Gadjah Mada, Sekip Utara, Yogyakarta 55281, Indonesia; ^6^Department of Chemistry and Biomolecular Science, Faculty of Engineering, Gifu University, Gifu 501-1193, Japan

## Abstract

The polyherbal formulation containing *Allium sativum* L., *Terminalia bellirica* (Gaertn.) Roxb., *Curcuma aeruginosa* Roxb., and *Amomum compactum* Sol ex. Maton has been used for hypertension treatment empirically. Our previous study showed its blood pressure-lowering effect on a rat model of hypertension. However, toxicity data were not available for this polyherbal formulation. This study is aimed at evaluating the acute and subchronic oral toxicity of the polyherbal formulation in rats. The acute toxicity study was conducted on 6 female Wistar rats using the fixed-dose method for the treatment group and 5 female Wistar rats for the control. The single dose of 2,000 mg/kg of the polyherbal formulation was given orally. There were no significant toxic effects and no death observed until the end of the study, and it was showed that the lethal dose 50% (LD50) of the polyherbal formulation was estimated to be more than 2,000 mg/kg. The macroscopic and microscopic examination of vital organs showed no symptoms of toxicity. At the subchronic toxicity study, the polyherbal formulation with 3 dose variations of 252 mg/kg, 1,008 mg/kg, and 4,032 mg/kg was administered for 91 days orally. The lowest dose of 252 mg/kg is equivalent to the daily recommended dose for a human. There were no significant toxic effects observed at all doses on physical sign and symptoms, weight gain, food intake, hematological parameters, biochemical parameters, and macroscopic and microscopic examination of organs. These findings showed that the short- and long-term oral administration of the polyherbal formulation is safe to use within its dose recommendation.

## 1. Introduction

Hypertension is a chronic disease that requires long-term medical treatment. Generally, antihypertensive works symptomatically to decrease blood pressure. The use of antihypertensive in the long term may cause various adverse side effects. Many researchers recommend a comprehensive approach in managing hypertension by diet regulation, exercise, and use of natural ingredients. Many medicinal plants grown in Indonesia have been traditionally used by communities to treat hypertension. Some plants have demonstrated antihypertensive activity on various hypertension animal models with various methods. These antihypertensive medicinal plants are *Phyllanthus niruri* [1], *Catharanthus roseus* [2], *Momordica charantia* [3], *Aloe vera* [4], *Morinda citrifolia* [5], *Centella asiatica* [6], and *Allium sativum* [7, 8]. *Nigella sativa* has been tested for hypertensive patients [9]. The antihypertensive mechanism of action of some plants has also been studied. *Alstonia scholaris* has been shown to have antihypertensive activity by inhibiting calcium ion channels [10].

The polyherbal formulation tablet containing *Allium sativum* L., *Terminalia bellirica* (Gaertn.) Roxb., *Curcuma aeruginosa* Roxb., and *Amomum compactum* Sol ex. Maton is indicated to treat hypertension. Nugrahaningsih et al. [11] reported that this polyherbal showed blood pressure-lowering effect on a rat model of hypertension. The ingredients of polyherbal formulation have been traditionally used by Indonesian people to treat cardiovascular disorders including hypertension. Some research results showed that the extract of each plant in this formulation has pharmacological activities that support its use in hypertension treatment.

*Allium sativum* or garlic was studied for cardiovascular disease. The meta-analysis study showed that garlic can lower the blood pressure of hypertensive patients but not for normal people [12]. Ried et al. [13] reported that garlic can lower blood pressure better than placebo. Garlic-derived polysulfides stimulate the production of the vascular gasotransmitter hydrogen sulfide (H_2_S) and enhance the regulation of endothelial nitric oxide (NO), which induce smooth muscle cell relaxation, vasodilation, and blood pressure reduction [8]. Garlic has been shown to have antihypertensive, antihyperlipidemic, antioxidant, and antiatherosclerotic effects [14–16]. *Terminalia bellirica* is shown to have antioxidant and antihypertensive effects [17]. *Curcuma aeruginosa* rhizoma and *Amomi fructus* (*Amomum compactum*) or cardamom have also been proven as antioxidants [18–20].

The development of traditional medicine requires both preclinical and clinical testing. One of the preclinical testing is toxicity test to ensure the safety of a formulation. Toxicity tests were performed to obtain data or information on the safety level of the compound in the animal. The oral acute toxicity test is a test performed on a compound that will be given orally. Acute toxicity tests are performed before further tests such as subchronic toxicity, teratogenic testing, mutagenesis testing, and carcinogenic testing. The oral subchronic toxicity test is a test to detect toxic effects after administration for a part of the animal's lifespan, but not more than 10% of animal life. The principle of the oral subchronic toxicity test is the tested compound formulation in several doses given daily in several groups of test animals with one dose per group for 28 or 91 days. The animal should be observed daily to determine the toxicity. Animals that die during observation are immediately autopsied; organ and tissue are observed macropathologically and histopathologically. At the end of the observation, all living animals were autopsied and macropathologically observed in each organ. Hematology, clinical biochemistry, and histopathology examinations were also performed. The purpose of the subchronic toxicity test is to obtain information on the toxic effects of undetectable substances on acute toxicity tests, information of possible toxic effects after repeated exposure of the testing compound for a certain period of time, and information of the dose that does not cause toxic effects (No Observed Adverse Effect Level (NOAEL)) and to study the cumulative effects and reversibility effects of the compound [21]. In this study, acute toxicity and oral subchronic toxicity studies of polyherbal formulation tablets were performed on the rat.

## 2. Materials and Methods

### 2.1. Polyherbal Formulation

The polyherbal formulation tablets were obtained from PT. Marguna Tarulata APK Farma, Indonesia. Each tablet of polyherbal formulation weighed 600 mg containing 180 mg bulbs of *Allium sativum* L., 60 mg fruit of *Terminalia bellirica* (Gaertn.) Roxb., 60 mg rhizome of *Curcuma aeruginosa* Roxb., 35 mg fruit of *Amomum compactum* Sol ex. Maton, and 265 mg of other materials. Tablets were powderized and made suspension with adding water. For maintaining the stability of the preparation, the only fresh suspension was given to the rat by oral gavage. The suspension was given with a volume of 10 mL/kg.

### 2.2. Experimental Animals

The Wistar rats were obtained from Animal House, Faculty of Medicine, Public Health and Nursing, Universitas Gadjah Mada, Yogyakarta, Indonesia. They were maintained in the room with 12 h light/dark cycle, 70% humidity, 23-25°C temperature, and sufficient ventilation and housed in cages covered with wire net. All animals were given access to food and water ad libitum. Experiments were conducted following the internationally accepted principles for animal laboratory use and care and approved by the Medical and Health Research Ethics Committee of Faculty of Medicine, Public Health and Nursing, Universitas Gadjah Mada–Sardjito General Hospital Yogyakarta.

### 2.3. Acute Oral Toxicity Study

The acute toxicity test was conducted according to OECD guideline 420 [22] using a fixed-dose procedure. The method provides information on the hazardous properties and allows the substance to be ranked and classified according to the Globally Harmonized System (GHS) for the classification of chemicals which cause acute toxicity. The OECD guidelines indicate that testing in one sex (usually females) is considered sufficient [22]. Six female Wistar rats 8-10 weeks old weighing 120 g ± 10 g were acclimatized for 7 days. The initial dose was chosen at 300 mg/kg with the consideration that there were no *in vivo* and *in vitro* toxicity data from the polyherbal formulation tablet. In the preliminary study on one rat with a dose of 300 mg/kg, we did not find any toxicity symptoms, so the dose was increased to 2,000 mg/kg. Further preliminary tests at a dose of 2,000 mg/kg also showed that there were no toxicity symptoms. Furthermore, in the main test, a dose of 2,000 mg/kg on 4 additional rats was continued. Each animal was observed and recorded for toxicity symptoms within the first 24 h, and observation was continued for 14 days. Then, the compound will be ranked and classified according to the Globally Harmonized System (GHS) for the classification of chemicals which cause acute toxicity [22].

### 2.4. Subchronic Oral Toxicity Study

The subchronic toxicity test was conducted according to the OECD guideline 408 for testing chemicals [23]. Eighty Wistar rats aged 8-10 weeks of both sexes were divided into 4 groups (*n* = 20, 10 males and 10 females), 3 treatment groups and 1 control group. The treatment groups were given polyherbal formulation 252, 1,008, and 4,032 mg/kg for each group. The control group was given water 10 mL/kg. The polyherbal formulation or water was given by oral gavage once daily at 7:00-8:00 am for 91 days. The rats were observed for physical signs and symptoms of the possibility of poisoning. The weight gain and food intake were calculated weekly. The laboratory examinations for hematological parameters and biochemical parameters were performed before treatment, after 45 days of treatment, and after 91 days of treatment. Macroscopic and histopathological examination for the vital organs was performed after 91 days of the study. Data were presented as mean ± SD. The mean difference of all groups was analyzed using one-way ANOVA continued by Tukey's multiple comparison test. The baseline hematology and clinical chemistry values for Charles River Wistar rats [24] were used as reference as normal values for results of hematological and biochemical examination.

## 3. Results

### 3.1. Acute Oral Toxicity Study

The observations of physical signs and symptoms and the possibility for toxic symptoms were performed on the first 30 minutes, 1 h, and 24 h and continued every day for 14 days. There were no signs of toxicity in the preliminary study with a dose of 300 mg/kg on 1 rat, also when the dose of 2,000 mg/kg was given to another 1 rat, and in the main testing of the same dose (2,000 mg/kg) in 4 rats.  Table 1 shows that the body weight of each rat before receiving polyherbal formulation, after 7 days, and after 14 days looks normal. The weight gain for week I and week II during the 14-day observations looks normal also.

**Figure 1:**
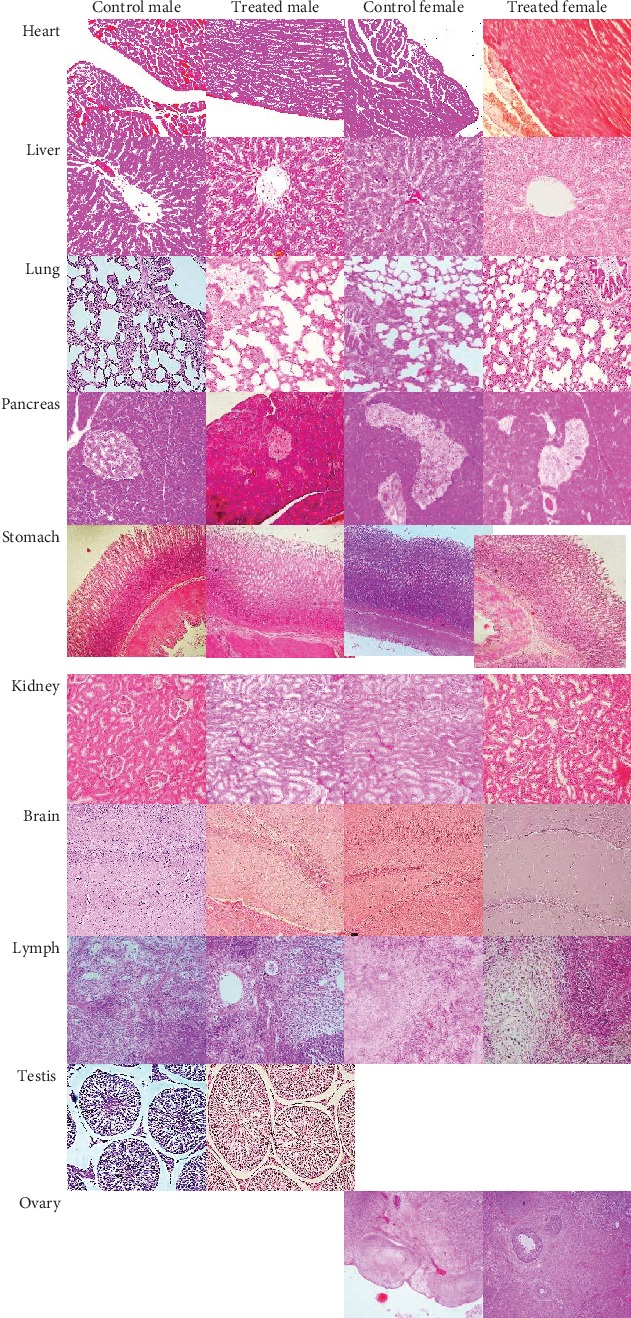
Effects of the 2,000 mg/kg polyherbal formulation on various rat organ histomorphology in the acute oral toxicity study (hematoxylin and eosin stained, magnification 400x).

There were no abnormalities found on macroscopic examination of the heart, liver, lung, stomach, pancreas, kidney, brain, lymph, and ovary.  Table 2 shows the absolute organ weight of rats in the acute toxicity study, and Table 3 shows the relative organ weight. Both the absolute organ weight and relative organ weight showed normal weight. In the microscopic examination, there was no abnormality found in the heart, liver, lung, pancreas, stomach, kidney, brain, lymph, testis, and ovary (Figure 1). Therefore, the polyherbal formulation tested showed that the lethal dose 50% (LD50) was more than 2,000 mg/kg. The Globally Harmonized System (GHS) classification criteria for acute toxicity suggested that this polyherbal formulation was classified in category 5 or unclassified (2,000 mg/kg < LD50 < 5,000 mg/kg) (OECD, 2001).

### 3.2. Subchronic Oral Toxicity Study

The results showed that there were no significant physical toxicity signs and symptoms in all rats in all groups. There were no symptoms of toxicity for each dose given, both male and female rats, and no death.

**Figure 2:**
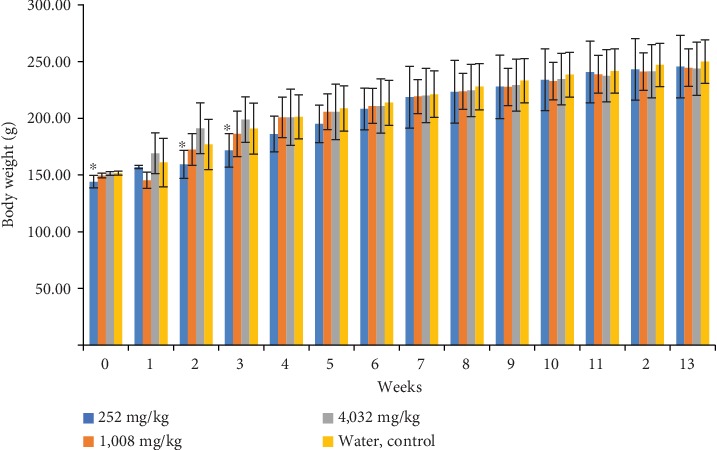
Mean of the body weight (g ± SD) weekly of male group rats which received polyherbal formulation or control for 13 weeks in 91 days in the subchronic oral toxicity study (^∗^*p* < 0.05, the significant difference compared to the control group using the one-way ANOVA continued by Tukey's multiple comparison test).

**Figure 3:**
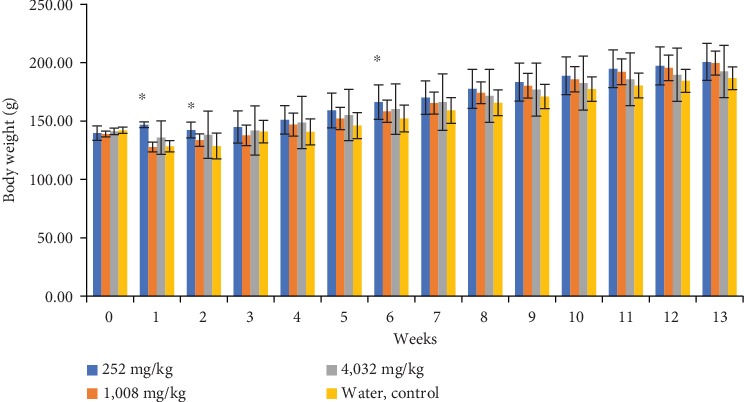
Mean of the body weight (g ± SD) weekly of female group rats which received polyherbal formulation or control for 13 weeks in 91 days in the subchronic oral toxicity study (^∗^*p* < 0.05, the significant difference compared to the control group using the one-way ANOVA continued by Tukey's multiple comparison test).

In male rat groups, the body weight of the rat group which received polyherbal formulation at a dose of 252 mg/kg looks lower (*p* < 0.05) than that of the control group in the 2^nd^ and 3^rd^ week. However, the differences were observed since week 0 (before the experiment). After the 3^rd^ week, the body weight generally had no significant difference in the control group (Figure 2). In female rat groups, the body weight of the rat group which received polyherbal formulation at a dose of 252 mg/kg was higher (*p* < 0.05) than that of the control group at the 1st, 2nd, and 6th week. The body weight of the rat group which received polyherbal formulation at a dose of 4,032 mg/kg was higher (*p* < 0.05) than that of the control group at the 1st week. However, there were generally no significant differences observed in body weight at the 3rd, 4th, 5th, 7th, 8th, 9th, 10th, 11th, 12th, and 13th week (Figure 3).

In male rat groups, the mean weight gain of rats in all groups which received polyherbal formulation at a dose of 252, 1,008, and 4,032 mg/kg at the 3rd, 5th, 6th, 7th, 9th, 10th, and 13th week did not show any difference (*p* > 0.05) compared to that in the control group (Table 4). Group I rats which received polyherbal formulation at a dose of 252 mg/kg showed lower weight gain (*p* < 0.05) than the control group at the 2nd week and 12th week and higher weight gain (*p* < 0.05) than the control group at the 11th week. Group II rats which received 1,008 mg/kg of polyherbal formulation showed decreasing body weight at the 1st week; however, the weight gain at the 2nd week was higher (*p* < 0.05) than the control group. At the 8th week, the weight gain of this group was lower (*p* < 0.05) than the control group; at the 11th week, the weight gain was higher (*p* < 0.05) than the control group, and at the 12th week, the weight gain was lower (*p* < 0.05) than the control group. Group III rats which received polyherbal formulation at a dose of 4,032 mg/kg showed lower weight gain (*p* < 0.05) than the control group at the 4th, 8th, and 12th week.

In female rat groups, the mean weight gain of rats in all groups which received polyherbal formulation at a dose of 252, 1,008, and 4,032 mg/kg at the 2nd, 7th, 9th, and 10th week did not show any difference (*p* > 0.05) compared to that in the control group (Table 4). Group I rats which received polyherbal formulation at a dose of 252 mg/kg showed higher weight gain (*p* < 0.05) than the control group at the 1st, 5th, 7th, 11th, and 13th week and lower weight gain (*p* < 0.05) than the control group at the 3rd and 12th week. Group II rats which received 1,008 mg/kg of polyherbal formulation showed lower weight gain at the 3rd week; however, the weight gain at the 4th, 8th, 11th, and 13th week was higher (*p* < 0.05) than the control group. Group III rats which received polyherbal formulation at a dose of 4,032 mg/kg showed lower weight gain (*p* < 0.05) than the control group at the 3rd and 6th week. These findings showed that sometimes the weight gain was higher or lower or has no difference compared to the control group showing that there was no tendency to decrease or increase weight gain due to the administration of the polyherbal formulation.

The mean food intake of male rats in all groups which received polyherbal formulation at a dose of 252, 1,008, and 4,032 mg/kg at the 1st, 3rd, 11th, 12th, and 13th week did not show any difference (*p* > 0.05) compared to that in the control group (Table 5). Group I rats which received polyherbal formulation at a dose of 252 mg/kg showed higher food intake (*p* < 0.05) than the control group at the 2nd week and 10th week and lower food intake (*p* < 0.05) than the control group at the 6th and 7th week. Group II rats which received 1,008 mg/kg of polyherbal formulation showed lower (*p* < 0.05) food intake than the control group at the 5th, 7th, 8th, and 9th week; however, the food intake at the 10th week was higher (*p* < 0.05) than the control group. Group III rats which received polyherbal formulation at a dose of 4,032 mg/kg showed lower food intake (*p* < 0.05) than the control group at the 7th week; however, the mean food intake was higher (*p* < 0.05) than the control group at the 10th and 12th week.

In female rat groups, the mean food intake of rats in all groups which received polyherbal formulation at a dose of 252, 1,008, and 4,032 mg/kg at the 2nd, 8th, and 10th week did not show any difference (*p* > 0.05) compared to that in the control group (Table 5). Group I rats which received polyherbal formulation at a dose of 252 mg/kg showed higher food intake (*p* < 0.05) than the control group at the 2nd, 8th, and 9th week and lower food intake (*p* < 0.05) than the control group at the 6th, 7th, and 11th week. Group II rats which received 1,008 mg/kg of polyherbal formulation showed lower food intake (*p* < 0.05) at the 4th and 5th week; however, the food intake at the 8th and 9th week was higher (*p* < 0.05) than the control group. Group III rats which received polyherbal formulation at a dose of 4,032 mg/kg showed lower food intake (*p* < 0.05) than the control group at the 5th and 13th week; however, the food intake was higher (*p* < 0.05) at the 8th and 9th week. These findings showed that sometimes the food intake was higher or lower or has no difference compared to the control group showing that there was no tendency to decrease or increase food intake due to the administration of the polyherbal formulation. When we observed the feces along the experiments, they showed to be normal soft feces in both treatment and control groups indicating neither diarrhea nor constipation.

Hematologic examinations for the hemoglobin level, total red blood cell (RBC), hematocrit, mean corpuscular volume (MCV), mean corpuscular hemoglobin (MCH), mean corpuscular hemoglobin concentration (MCHC), total white blood cell (WBC), eosinophils, basophils, band neutrophils, segmented neutrophil, lymphocytes, and monocytes in WBC differential are presented at Tables 6–8. The hematological parameters of male group rats before receiving polyherbal formulation or control showed that the MCH value of the group receiving polyherbal formulation at a dose of 1,008 mg/kg was higher (*p* < 0.05) than that of the control group and that the total WBC of the group receiving polyherbal formulation at a dose of 252 mg/kg was lower (*p* < 0.05) than that of the control group. However, both values of the MCH (20.06 ± 0.46) and the total WBC (4, 600 ± 812.40) were normal values [24]. The other hematological parameters in other treatment group rats have normal value, and there were no significant differences with the control group. The hematological parameters of female group rats showed that the MCH value of the group receiving polyherbal formulation at a dose of 252 mg/kg was lower (*p* < 0.05) than that of the control group, and the group which received 1,008 mg/kg of polyherbal formulation was higher than the control group. However, both values of MCH were normal values according to Charles River Laboratories [24] (Table 6).

The hematological parameters were performed after 45 days of treatment, for both male and female for all treatment groups, showing that there were no significant differences in the control group (Table 7). All hematological parameters showed normal values [24].

The hematological parameters of both male and female rat groups after 91 days of treatment with polyherbal formulation or control are presented in Table 8. In the male group, the mean of total RBC of the group receiving polyherbal formulation at a dose of 4,032 mg/kg (7.44 ± 0.18) was lower (*p* < 0.05) than that of the control group (8.46 ± 0.27). The mean of MCH of the group receiving polyherbal formulation at a dose of 4,032 mg/kg (20.90 ± 1.11) was higher than that of the control group (19.56 ± 0.46). However, both the mean total of RBC and the mean of MCH were within the normal range. The hematological parameters of female group rats showed that the mean total of RBC of the group which received polyherbal formulation at a dose of 252 mg/kg (8.12 ± 0.47) and (7.37 ± 0.30) of the group which received polyherbal formulation at a dose of 4,032 mg/kg was higher (*p* < 0.05) than that of the control group (6.61 ± 0.65). The mean of hematocrit of all three group treatments was higher (*p* < 0.05) than that of the control group. The mean of MCHC of two groups which received polyherbal formulation at a dose of 252 mg/kg and 1,008 mg/kg was lower (*p* < 0.05) than that of the control group. However, the significant difference value of these groups to the control group was within the normal range.

The blood examinations of the biochemical parameters for urea, creatinine, total protein, albumin, globulin, aspartate aminotransferase (AST) or glutamic-oxaloacetic transaminase (GOT), and alanine aminotransferase (ALT) or glutamic-pyruvic transaminase (GPT) are presented at Tables 9–11.  Table 9 shows that all biochemical parameters before the rats received polyherbal formulation in all three treatment groups were not different (*p* > 0.05) compared to those in the control group.

The examination of renal function during the subchronic toxicity test was conducted by examining the urea and creatinine levels of plasma. The mean of the urea level of female group rats after receiving polyherbal formulation for 45 days was lower (*p* < 0.05) than that of the control group; however, the value was within the normal range (Table 10). The other treatment groups both males and females, after 45 days and after 91 days, showed that there were no differences (*p* > 005) in the mean of urea and creatinine levels (Tables 10 and 11). These findings showed that administration of polyherbal formulation on rats at a dose to 4,032 mg/kg daily for 91 days gives no abnormal renal function.

The examination of liver function during the subchronic toxicity test was conducted by examining the level of the total protein, albumin, globulin, AST, and ALT (Tables 9–11). Both rat male and female groups which received polyherbal formulation at a dose of 252 mg/kg showed a lower (*p* < 0.05) mean of globulin than the control group after 45 days of treatments, although these results are within the normal range (Table 10). The mean of AST of male group rats was higher (*p* < 0.05) than that of the normal group (Table 10); however, it was not different (*p* > 005) with the mean AST before treatment (Table 9). After 91 days of treatments, the mean of AST was not different (*p* > 0.05) from the control group (Table 11). The female group which received polyherbal formulation at a dose of 1,008 mg/kg showed the lower mean of urea (*p* < 0.05) than the control group, although these results are within the normal range (Table 10). The mean urea showed no difference (*p* > 0.05) with the control groups after 91 days of treatment (Table 11). Both male and female groups which received polyherbal formulation at a dose of 4,032 mg/kg after 45 days showed the higher (*p* < 0.05) mean of total protein and globulin than the control group, although these results are within the normal range (Table 10). The mean of total protein and globulin showed no difference (*p* > 0.05) in the control groups after 91 days of treatment (Table 11). These findings showed that administration of polyherbal formulation at a dose of 4,032 mg/kg daily for 91 days showed no abnormal liver function.

The macroscopic examination after 91 days of treatments showed no organ abnormalities in all treatment and control groups in both male and female groups. Both male and female groups showed that the absolute and relative organ weight of the heart, liver, lung, stomach, intestine, kidney, brain, spleen, testis, and ovary in all treatment groups did not show any significant differences (*p* > 0.05) compared to the control group (Tables 12 and 13). These results indicated that administration of polyherbal formulation tablets at doses up to 4,032 mg/kg once daily for 91 consecutive days did not result in changes in absolute weight or relative weight of the heart, liver, lung, stomach, intestine, kidney, brain, spleen, testes, and ovaries.

**Figure 4:**
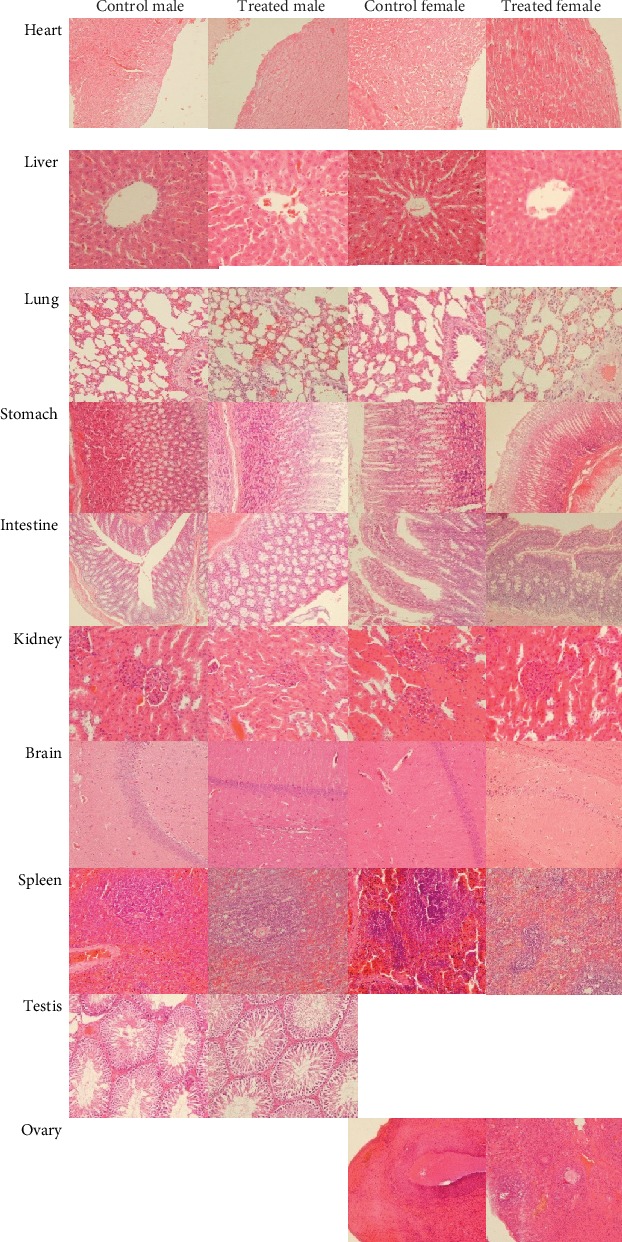
The histomorphology of rats which received 4,032 mg/kg polyherbal formulation or control after 91 days in the subchronic oral toxicity study (hematoxylin and eosin stained, magnification 400x).

To perform the microscopic examination to the organs, each organ was prepared for histopathological examination. The light microphotographs of the transverse section of vital organs from the subchronic oral toxicity study of the polyherbal formulation and the control group rats are shown in Figure 4. Histopathological examination under a light microscope using multiple magnification powers of the treatment and control groups of rats showed the normal structure, and there is no alteration in the cell structure or any unfavorable effect in organs. These findings suggested that administration of polyherbal formulation tablets at doses up to 4,032 mg/kg body weight once daily for 91 consecutive days has no toxic effect on the analyzed organs.

## 4. Discussion

The polyherbal formulation containing *Allium sativum* L., *Terminalia bellirica* (Gaertn.) Roxb., *Curcuma aeruginosa* Roxb., and *Amomum compactum* Sol ex. Maton is indicated to help lower blood pressure. This polyherbal formulation tablet has been studied and proven to decrease blood pressure as indicated. Three weeks of treatment with 126 mg/kg of the polyherbal on the rat model of hypertension showed lower systolic blood pressure than the control group [11]. Each ingredient in polyherbal formulation tablets has also been reported by researchers to have activity such as antihypertensive, antihyperlipidemic, antisclerotic, and antioxidant. *Allium sativum* or garlic has been known to have activity as antihypertensive [7, 8, 14, 15, 25, 26], antihyperglycemic, antihyperlipidemic, anti-inflammatory [27], and antihypercholesterolemia [28]. *Allium sativum*-derived polysulfides stimulate the production of the vascular gasotransmitter hydrogen sulfide (H_2_S) and enhance the regulation of endothelial nitric oxide (NO), which induce smooth muscle cell relaxation, vasodilation, and blood pressure reduction [8]. Hydrogen sulfide (H_2_S) is an endogenous gasotransmitter, which regulates the homeostasis of cardiovascular, respiratory, gastroenteric, and central nervous systems and modulates the inflammatory process. Impaired biosynthesis of endogenous H_2_S is associated with many important diseases. Drugs acting as sources of exogenous H_2_S, consequently, show promise as treatments for serious disorders, including hypertension [29]. The growing evidence about a protective role exhibited by H_2_S against myocardial ischemia/reperfusion (I/R) led to an increasing interest for the possible mechanism of action accounting for the H_2_S cardioprotective effect and to the discovery of the involvement of several targets [30]. However, other studies showed that in normotensive rats, the vascular actions of H_2_S required nitric oxide and vice versa. In contrast, in spontaneously hypertensive rats, the H_2_S-induced actions scarcely depend on nitric oxide release; also, the nitric oxide effects are largely H_2_S-independent and represent the first step for understanding pathophysiological mechanisms of nitric oxide/H_2_S interplays under both normotensive and hypertensive conditions [31]. *Terminalia bellirica* is also shown to have antihypertensive [17] and antioxidant [32] activity. *Curcumae aeruginosae* known by the local name of black temu and *Amomi fructus* (cardamom) have been proven to have antioxidant activity [18–20]. Curcumin is one of the active compounds of *Curcumae aeruginosae* [33]. Rachmawati et al. [34] reported that curcumin showed *in vitro* antihypertensive activity. However, other studies showed low intestinal absorption of curcumin. The reason for the low bioavailability of oral curcumin may be related to its low solubility and the efflux effect of P-gp and MRP2 in intestinal epithelial cells during intestinal absorption [35]. In this toxicity study, we did not observe the curcumin absorption.

In the present acute toxicity study, polyherbal formulation tablets up to 2,000 mg/kg did not cause toxicity of the treated rats, either in a preliminary trial with one rat or in the primary study with 4 rats. According to OECD [22], the use of an additional upper fixed-dose level of 5000 mg/kg should only be considered when there is a strong likelihood that results of such a test have a direct relevance for protecting humans, animal health, or environment. When the main study at a dose of 2,000 mg/kg showed no toxicity symptoms, the formulation can be categorized as unclassified material. In this present acute toxicity study, the LD50 value could not be determined because the largest dose recommended in the study (2,000 mg/kg) did not show any toxicity symptoms. It is therefore assumed that the LD50 value of polyherbal formulation tablets in this study was more than 2,000 mg/kg. Thus, it can be concluded from this study that the polyherbal formulation tablets were included in the unclassified criteria.

As a continuation of the acute toxicity study, this oral subchronic toxicity study was conducted on Wistar rats. Polyherbal formulation tablets were administered once daily for 91 consecutive days. According to the OECD [21], the oral subchronic toxicity study is a test to detect toxic effects that occurred after oral administration of testing materials in animals for a part of the animal's lifetime, but not more than 10% of their lifetime. This subchronic toxicity study showed that the administration of polyherbal formulation tablets in rats up to the maximum dose that technically can still be given to white rats, i.e., 4,032 mg/kg, did not cause toxicity symptoms or death. From the physical examination, macroscopic or histopathology of internal organs also found no toxic effects or adverse effects after administration of polyherbal formulation tablets at a dose up to 4,032 mg/kg. The results of this examination support the survival data obtained indicating that polyherbal formulation tablets were not harmful. The administration of polyherbal formulation tablets at a dose up to 4,032 mg/kg in rats for 91 consecutive days did not present any signs of toxicity, either on clinical observation, laboratory and macroscopic examination of internal organs, or microscopically.

## 5. Conclusions

The polyherbal formulation tablets were included in the unclassified criteria, with the LD50 value more than 2,000 mg/kg. The administration at a dose up to 4,032 mg/kg once daily for 91 consecutive days on subchronic toxicity study did not result in death and did not cause toxic effect symptoms that could be observed based on clinical, laboratory, macroscopic, and microscopic examination.

## Figures and Tables

**Table 1:** The rat body weight before and after receiving polyherbal formulation at a single dose of 300 mg/kg (*n* = 1), 2,000 mg/kg (*n* = 5), and control (*n* = 5) for 14-day observations in the acute oral toxicity study.

Dose (mg/kg)	No. of rats	Body weight (g) before and after receiving polyherbal formulation			Weight gain	
		Before	7 days after	14 days after	Week I (%)	Week II (%)
Control	1	130	135	138	3.85	2.22
	2	130	134	137	3.08	2.24
	3	129	133	138	3.10	3.76
	4	129	135	140	4.65	3.70
	5	127	132	139	3.94	5.30
	Mean ± SD	129.00 ± 1.22	133.80 ± 1.30	138.40 ± 1.14	3.72 ± 0.66	3.45 ± 1.28

300	1	130	135	144	3.85	6.67

2,000	1	126	132	138	4.76	4.55
	2	126	130	136	3.17	4.62
	3	130	134	137	3.08	2.24
	4	128	134	138	4.69	2.99
	5	129	133	137	3.10	3.01
	Mean ± SD	127.80 ± 1.79	132.60 ± 1.67	137.20 ± 0.84	3.76 ± 0.88	3.48 ± 1.05

**Table 2:** The absolute organ weight of rats which received polyherbal formulation at a dose of 2,000 mg/kg and control in the acute oral toxicity study.

Dose (mg/kg)	Organ	Absolute organ weight (g) of rat no.					Means	SD
		1	2	3	4	5		
2,000	Heart	0.50	0.54	0.53	0.52	0.53	0.52	0.02
	Liver	3.90	3.30	3.19	3.90	3.78	3.61	0.34
	Lung	1.01	1.04	1.03	1.03	1.05	1.03	0.01
	Stomach	1.39	1.32	1.42	1.39	1.28	1.36	0.06
	Pancreas	0.57	0.63	0.57	0.59	0.60	0.59	0.02
	Kidney	0.57	0.63	0.57	0.59	0.57	0.59	0.03
	Brain	1.44	1.45	1.46	1.45	1.44	1.45	0.01
	Lymph	0.32	0.29	0.29	0.33	0.30	0.31	0.02
	Ovary	0.34	0.36	0.37	0.34	0.33	0.35	0.02

Control	Heart	0.57	0.57	0.56	0.59	0.60	0.58	0.02
	Liver	3.50	3.49	3.41	3.40	3.50	3.46	0.05
	Lung	1.19	1.20	1.17	1.16	1.17	1.18	0.02
	Stomach	1.27	1.34	1.27	1.26	1.25	1.28	0.04
	Pancreas	0.59	0.80	0.60	0.69	0.63	0.66	0.09
	Kidney	0.61	0.59	0.58	0.57	0.55	0.58	0.02
	Brain	1.29	1.27	1.25	1.28	1.30	1.28	0.02
	Lymph	0.37	0.36	0.35	0.33	0.34	0.35	0.02
	Ovary	0.35	0.31	0.33	0.34	0.36	0.34	0.02

**Table 3:** The relative organ weight of rats 14 days after receiving polyherbal formulation at a dose of 2,000 mg/kg and control in the acute oral toxicity study.

Dose (mg/kg)	Organ	Relative organ weight (%) of rat no.					Means	SD
		1	2	3	4	5		
2,000	Heart	0.36	0.40	0.39	0.38	0.39	0.38	0.01
	Liver	2.83	2.43	2.33	2.83	2.76	2.63	0.24
	Lung	0.73	0.76	0.75	0.75	0.77	0.75	0.01
	Stomach	1.01	0.97	1.04	1.01	0.93	0.99	0.04
	Pancreas	0.41	0.46	0.42	0.43	0.44	0.43	0.02
	Kidney	0.41	0.46	0.42	0.43	0.42	0.43	0.02
	Brain	1.04	1.07	1.07	1.05	1.05	1.06	0.01
	Lymph	0.23	0.21	0.21	0.24	0.22	0.22	0.01
	Ovary	0.25	0.26	0.27	0.25	0.24	0.25	0.01

Control	Heart	0.41	0.42	0.41	0.42	0.43	0.42	0.01
	Liver	2.54	2.55	2.47	2.43	2.52	2.50	0.05
	Lung	0.86	0.88	0.85	0.83	0.84	0.85	0.02
	Stomach	0.92	0.98	0.92	0.90	0.90	0.92	0.01
	Pancreas	0.43	0.58	0.43	0.49	0.45	0.48	0.06
	Kidney	0.44	0.43	0.42	0.41	0.39	0.41	0.02
	Brain	0.93	0.93	0.91	0.91	0.94	0.92	0.01
	Lymph	0.27	0.26	0.25	0.24	0.24	0.25	0.01
	Ovary	0.25	0.23	0.24	0.24	0.26	0.24	0.01

**Table 4:** Mean of the weight gain (g ± SD) weekly of group rats which received polyherbal formulation or control for 13 weeks in 91 days in the subchronic oral toxicity study.

Group and dose (*n* = 10)		Weight gain (g) weekly												
		1	2	3	4	5	6	7	8	9	10	11	12	13
Male														
I (252 mg/kg)	Mean	12.90	2.40	12.40	14.40	8.90	13.10	10.40	4.80	4.40	6.20	6.80	2.40	2.30
	SD	6.31	12.14	4.50	7.06	2.77	18.37	11.92	1.99	2.72	1.14	1.32	0.70	1.16
	*p*	0.591	0.007^∗^	0.815	0.303	0.286	0.060	0.380	0.064	0.305	0.025	0.0001^∗^	0.0001^∗^	0.137
II (1,008 mg/kg)	Mean	-4.30	27.10	13.90	14.50	5.00	5.10	8.30	4.60	3.80	5.20	6.00	2.40	3.40
	SD	6.38	8.66	11.25	6.29	2.67	0.88	5.77	2.63	2.30	0.42	1.05	1.17	1.26
	*p*	0.048^∗^	0.022^∗^	0.988	0.291	0.128	1.000	0.825	0.040^∗^	0.105	1.000	0.0001^∗^	0.0001^∗^	0.390
III (4,032 mg/kg)	Mean	17.90	22.10	7.50	2.10	4.80	5.20	9.20	4.40	4.70	5.30	3.00	4.00	2.20
	SD	18.38	13.47	27.35	12.06	2.15	0.79	4.34	1.96	1.16	1.25	0.94	1.33	0.79
	*p*	0.204	0.195	0.346	0.037^∗^	0.099	0.981	0.615	0.024^∗^	0.471	0.816	0.546	0.024^∗^	0.091
IV (water, control)	Mean	9.30	15.90	14.00	10.40	7.30	5.10	7.60	6.50	5.40	5.20	3.30	5.30	3.00
	SD	21.57	6.03	5.56	7.59	4.92	0.74	2.07	1.08	2.12	0.79	1.06	1.57	0.82

Female														
I (252 mg/kg)	Mean	5.36	-4.50	2.50	6.20	8.10	7.10	3.80	7.50	5.80	5.50	5.90	2.60	3.30
	SD	6.84	6.82	12.31	15.50	4.28	1.79	13.20	3.10	1.32	1.18	0.99	0.97	1.49
	*p*	0.0001^∗^	0.173	0.006^∗^	0.141	0.016^∗^	0.082^∗^	0.326	0.317	0.873	0.206	0.0001^∗^	0.031^∗^	0.069^∗^
II (1,008 mg/kg)	Mean	-10.48	5.90	4.20	9.20	5.20	6.20	7.00	8.80	6.00	5.60	6.40	3.40	4.00
	SD	5.44	3.51	5.73	3.88	1.03	1.14	1.89	2.30	1.76	1.51	0.84	0.97	1.56
	*p*	0.705	0.100	0.020^∗^	0.033^∗^	0.852	0.859	0.975	0.025^∗^	0.873	0.267	0.0001^∗^	0.344	0.003^∗^
III (4,032 mg/kg)	Mean	-3.81	2.40	3.70	6.80	6.40	5.10	6.00	5.40	5.30	5.60	3.30	3.80	2.90
	SD	15.48	8.34	5.23	1.81	1.26	0.99	3.68	1.90	1.34	1.51	1.06	1.87	0.88
	*p*	0.061	0.511	0.014^∗^	0.108	0.353	0.082^∗^	0.774	0.272	0.206	0.267	0.650	0.751	0.208
IV (water, control)	Mean	-12.12	0.20	12.30	-0.20	5.40	6.10	6.90	6.50	5.40	6.30	3.10	4.00	2.30
	SD	7.18	10.00	3.13	10.11	1.26	0.88	1.66	0.97	0.97	1.34	0.99	1.56	0.48

^∗^ *p* < 0.05, the significant difference compared to the control group using the one-way ANOVA continued by Tukey's multiple comparison test.

**Table 5:** Mean of the food intake (g ± SD) weekly of group rats which received polyherbal formulation or control for 13 weeks in 91 days in the subchronic oral toxicity study.

Group and dose (*n* = 10)		Food intake (g) weekly												
		1	2	3	4	5	6	7	8	9	10	11	12	13
Male														
I (252 mg/kg)	Mean	76.50	85.00	86.00	110.00	109.00	95.00	108.00	112.00	116.00	120.00	116.50	118.00	117.50
	SD	6.69	5.27	5.16	9.43	5.68	5.27	7.53	5.87	4.59	0.00	4.12	4.22	4.25
	*p*	0.213	0.0001^∗^	0.267	0.777	0.518	0.0001^∗^	0.0001^∗^	0.129	0.820	0.0001^∗^	0.133	0.428	1.000
II (1,008 mg/kg)	Mean	75.50	76.00	86.00	87.00	96.00	101.00	104.00	107.00	110.00	120.00	118.00	120.00	118.50
	SD	4.38	4.59	5.16	4.22	6.99	4.59	3.94	5.37	4.08	0.00	2.58	0.00	2.42
	*p*	0.100	0.163	0.267	0.0001^∗^	0.001^∗^	0.207	0.0001^∗^	0.001^∗^	0.005^∗^	0.0001^∗^	0.703	0.021	0.496
III (4,032 mg/kg)	Mean	77.50	75.00	86.50	108.00	108.50	106.00	113.00	115.50	118.00	119.00	119.0	117.00	116.50
	SD	5.89	5.27	4.74	7.89	7.47	3.94	6.75	4.97	6.32	2.11	2.11	2.58	3.37
	*p*	0.404	0.349	0.373	0.398	0.028	0.207	0.033^∗^	1.000	0.495	0.003^∗^	0.703	1.000	0.496
IV (water, control)	Mean	79.50	73.00	88.50	111.00	107.00	103.50	118.50	115.50	116.50	115.50	118.50	117.00	117.50
	SD	3.69	3.50	4.74	8.76	7.15	3.37	2.42	3.69	4.12	4.38	2.42	2.58	2.64

Female														
I (252 mg/kg)	Mean	76.00	86.00	84.00	116.00	114.00	95.00	108.00	114.00	119.00	118.50	115.00	120.50	117.00
	SD	5.68	5.16	5.16	5.16	6.99	5.27	6.75	4.59	6.15	2.42	4.08	3.69	2.58
	*p*	0.844	0.0001^∗^	0.445	0.152	0.771	0.041^∗^	0.003^∗^	0.0001^∗^	0.0001^∗^	0.590	0.044^∗^	0.068	1.000
II (1,008 mg/kg)	Mean	79.00	75.00	81.50	84.00	90.00	120.50	109.00	114.50	117.00	120.00	120.00	117.50	118.00
	SD	6.58	5.27	6.69	4.59	9.43	39.33	3.16	2.84	3.50	0.00	0.00	4.25	2.58
	*p*	0.328	0.656	0.091	0.0001^∗^	0.0001^∗^	0.472	0.011	0.0001^∗^	0.001^∗^	0.285	0.172	1.000	0.404
III (4,032 mg/kg)	Mean	77.00	72.00	85.50	106.50	108.00	103.00	116.00	117.50	119.50	118.00	117.00	117.00	114.50
	SD	5.87	4.22	5.99	6.69	7.89	2.58	4.59	3.54	3.69	2.58	4.22	3.50	2.84
	*p*	0.844	0.080	0.848	0.052	0.048^∗^	0.227	0.656	0.0001^∗^	0.0001^∗^	0.285	0.490	0.756	0.042^∗^
IV (water, control)	Mean	76.50	76.00	86.00	112.00	115.00	114.00	115.00	106.50	110.00	119.00	118.00	117.50	117.00
	SD	4.12	5.16	5.16	7.53	5.77	4.59	4.71	3.37	3.33	2.11	2.58	2.64	2.58

^∗^ *p* < 0.05, the significant difference compared to the control group using the one-way ANOVA continued by Tukey's multiple comparison test.

**Table 6:** Mean of the hematological parameters of group rats before receiving polyherbal formulation or control in the subchronic oral toxicity study.

	Unit	Control	Dose (mg/kg)		
			252	1,008	4,032
Male (*n* = 5)					
Hemoglobin	g/dL	14.60 ± 0.92	13.78 ± 1.63	14.42 ± 1.33	15.16 ± 0.56
Total RBC	10^6^/cmm	7.75 ± 0.32	6.83 ± 1.24	7.18 ± 0.59	7.6 ± 0.47
Hematocrit	%	42.76 ± 1.67	38.46 ± 5.75	39.96 ± 3.99	42.22 ± 1.32
MCV	fL/red cell	55.24 ± 2.20	56.64 ± 3.05	55.60 ± 1.70	56.18 ± 2.78
MCH	pg	18.86 ± 1.27	20.44 ± 1.96	20.06 ± 0.46^∗^	20.12 ± 1.05
MCHC	g/dL	34.12 ± 1.04	35.98 ± 1.70	36.10 ± 0.48	35.88 ± 0.29
Total WBC	/cmm	7,800 ± 2,131.90	4,600 ± 812.40^∗^	9,080 ± 960.21	8,200 ± 595.82
Eosinophil	%	0 ± 0	0 ± 0	0 ± 0	0 ± 0
Basophil	%	0 ± 0	0 ± 0	0 ± 0	0 ± 0
Band neutrophil	%	0 ± 0	0 ± 0	0 ± 0	0 ± 0
Segmented neutrophil	%	32.00 ± 6.82	30.20 ± 12.52	22.4 ± 3.29	26.20 ± 4.21
Lymphocytes	%	68.00 ± 6.82	69.80 ± 12.52	77.20 ± 3.63	73.80 ± 4.21
Monocytes	%	0 ± 0	0 ± 0	0 ± 0	0 ± 0
Platelet	/cmm	974,200 ± 139,372.52	1,056,800 ± 79,722,64	1,029,200 ± 186,878.84	1,010,200 ± 182,066.47

Female (*n* = 5)					
Hemoglobin	g/dL	14.22 ± 1.37	13.38 ± 0.62	13.38 ± 0.62	13.58 ± 1.03
Total RBC	10^6^/cmm	6.90 ± 0.61	6.50 ± 0.43	6.50 ± 0.43	6.46 ± 0.28
Hematocrit	%	40.70 ± 4.79	35.78 ± 1.93	35.78 ± 1.93	36.10 ± 2.41
MCV	fL/red cell	58.82 ± 1.96	55.10 ± 1.29	55.01 ± 1.29	55.86 ± 2.20
MCH	pg	20.58 ± 0.28	20.36 ± 0.36^∗^	20.62 ± 0.48^∗^	21.04 ± 1.16
MCHC	g/dL	35.00 ± 0.78	37.42 ± 0.40	37.42 ± 0.40	35.62 ± 4.38
Total WBC	/cmm	9,400 ± 3,055.32	8,760 ± 1517.56	9,200 ± 1,665.83	7,500 ± 1,537.86
Eosinophil	%	0 ± 0	0 ± 0	0 ± 0	0 ± 0
Basophil	%	0 ± 0	0 ± 0	0 ± 0	0 ± 0
Band neutrophil	%	0 ± 0	0 ± 0	0 ± 0	0 ± 0
Segmented neutrophil	%	25.80 ± 18.70	23.80 ± 5.07	30.80 ± 3.35	25.60 ± 7.09
Lymphocytes	%	74.20 ± 18.70	76.20 ± 5.07	69.20 ± 3.35	74.40 ± 7.09
Monocytes	%	0 ± 0	0 ± 0	0 ± 0	0 ± 0
Platelet	/cmm	1,058,000 ± 93,706.99	813,200 ± 256,221	1,084,200 ± 275,366.48	1,078,800 ± 157,399.81

^∗^ *p* < 0.05, the significant difference compared to the control group using the one-way ANOVA continued by Tukey's multiple comparison test.

**Table 7:** Mean of the hematological parameters of group rats which received polyherbal formulation or control after 45 days in the subchronic oral toxicity study.

	Unit	Control	Dose (mg/kg)		
			252 mg/kg	1,008 mg/kg	4,032 mg/kg
Male (*n* = 5)					
Hemoglobin	g/dL	15.20 ± 1.57	16.46 ± 0.89	16.34 ± 0.80	15.76 ± 1.74
Total RBC	10^6^/cmm	8.32 ± 0.82	8.93 ± 0.46	8.98 ± 0.38	8.35 ± 1.25
Hematocrit	%	44.42 ± 5.61	48.66 ± 2.43	48.04 ± 1.65	45.70 ± 5.70
MCV	fL/red cell	53.26 ± 1.72	54.48 ± 1.58	53.54 ± 1.69	54.94 ± 2.22
MCH	pg	18.28 ± 0.84	18.46 ± 1.21	18.22 ± 1.14	18.96 ± 0.97
MCHC	g/dL	34.30 ± 1.39	33.82 ± 1.31	34.02 ± 1.14	34.56 ± 0.56
Total WBC	/cmm	8,220 ± 2,630.02	9,400 ± 3,143.25	8,800 ± 871.78	8,820 ± 683.37
Eosinophil	%	0 ± 0	0 ± 0	0 ± 0	0 ± 0
Basophil	%	0 ± 0	0 ± 0	0 ± 0	0 ± 0
Band neutrophil	%	0 ± 0	0 ± 0	0 ± 0	0 ± 0
Segmented neutrophil	%	27.00 ± 8.94	22.6 ± 4.22	23.80 ± 0.84	24.40 ± 4.77
Lymphocytes	%	75.00 ± 11.18	77.40 ± 4.22	76.20 ± 0.84	75.60 ± 4.77
Monocytes	%	0 ± 0	0 ± 0	0 ± 0	0 ± 0
Platelet	/cmm	746,400 ± 104,236.75	605,800 ± 277,151.94	915,400 ± 134,168.92	875,400 ± 138,833.71

Female (*n* = 5)					
Hemoglobin	g/dL	14.80 ± 0.45	14.60 ± 1.97	14.52 ± 0.70	14.52 ± 0.60
Total RBC	10^6^/cmm	7.61 ± 0.24	7.88 ± 0.98	7.61 ± 0.79	7.52 ± 0.56
Hematocrit	%	42.46 ± 1.52	42.64 ± 5.30	46.20 ± 13.78	41.02 ± 2.87
MCV	fL/red cell	55.78 ± 0.50	54.14 ± 0.47	53.70 ± 1.14	54.62 ± 2.35
MCH	pg	19.44 ± 0.21	18.52 ± 0.45	19.44 ± 1.25	19.38 ± 1.16
MCHC	g/dL	34.84 ± 0.44	34.20 ± 0.75	36.18 ± 1.57	35.46 ± 1.22
Total WBC	/cmm	6,940 ± 1,553.38	7,900 ± 1,759.26	9,060 ± 1,709.67	8,600 ± 1,935.20
Eosinophil	%	0 ± 0	0 ± 0	0 ± 0	0 ± 0
Basophil	%	0 ± 0	0 ± 0	0 ± 0	0 ± 0
Band neutrophil	%	0 ± 0	0 ± 0	0 ± 0	0 ± 0
Segmented neutrophil	%	20.20 ± 8.56	27.80 ± 6.98	23.40 ± 2.97	23.60 ± 8.08
Lymphocytes	%	79.80 ± 8.56	72.20 ± 6.98	76.60 ± 2.97	76.40 ± 8.08
Monocytes	%	0 ± 0	0 ± 0	0 ± 0	0 ± 0
Platelet	/cmm	731,200 ± 66,024.24	991,600 ± 209,812.54	966,400 ± 151,168.12	899,200 ± 149,032.55

^∗^ *p* < 0.05, the significant difference compared to the control group using the one-way ANOVA continued by Tukey's multiple comparison test.

**Table 8:** Mean of the hematological parameters of group rats which received polyherbal formulation or control after 91 days in the subchronic oral toxicity study.

	Unit	Control	Dose		
			252 mg/kg	1,008 mg/kg	4,032 mg/kg
Male (*n* = 5)					
Hemoglobin	g/dL	16.54 ± 0.71	16.00 ± 1.19	15.74 ± 0.63	15.54 ± 0.66
Total RBC	10^6^/cmm	8.46 ± 0.27	8.21 ± 0.41	7.91 ± 0.41	7.44 ± 0.18^∗^
Hematocrit	%	43.68 ± 1.86	43.72 ± 2.48	42.24 ± 2.62	40.50 ± 1.43
MCV	fL/red cell	51.60 ± 1.12	53.22 ± 1.02	53.38 ± 0.79	54.46 ± 2.40
MCH	pg	19.56 ± 0.46	19.50 ± 1.47	19.90 ± 0.72	20.90 ± 1.11^∗^
MCHC	g/dL	37.86 ± 0.63	36.64 ± 2.54	37.34 ± 1.66	38.36 ± 0.57
Total WBC	/cmm	9, 700 ± 3, 129.70	6, 060 ± 1, 244.19	9, 620 ± 1, 734.07	7, 020 ± 1930.54
Eosinophil	%	0 ± 0	0 ± 0	0 ± 0	0 ± 0
Basophil	%	0 ± 0	0 ± 0	0 ± 0	0 ± 0
Band neutrophil	%	0 ± 0	0 ± 0	0 ± 0	0 ± 0
Segmented neutrophil	%	33.60 ± 7.57	28.80 ± 8.70	34.20 ± 6.30	29.40 ± 9.56
Lymphocytes	%	66.40 ± 7.57	71.20 ± 8.70	65.80 ± 6.30	70.60 ± 9.56
Monocytes	%	0 ± 0	0 ± 0	0 ± 0	0 ± 0
Platelet	/cmm	905, 000 ± 127, 736.00	1, 000, 000 ± 138, 181.04	925, 600 ± 115, 248.86	892, 600 ± 195, 578.12

Female (*n* = 5)					
Hemoglobin	g/dL	13.84 ± 1.39	15.92 ± 0.59	14.44 ± 0.67	14.63 ± 2.22
Total RBC	10^6^/cmm	6.61 ± 0.65	8.12 ± 0.47^∗^	7.37 ± 0.38	7.37 ± 0.30^∗^
Hematocrit	%	35.12 ± 3.37	43.42 ± 2.63^∗^	40.20 ± 1.28^∗^	39.83 ± 3.61^∗^
MCV	fL/red cell	53.20 ± 1.84	53.48 ± 0.75	54.54 ± 1.27	53.95 ± 3.06
MCH	pg	20.96 ± 0.30	19.62 ± 0.43	19.62 ± 0.98	19.78 ± 2.44
MCHC	g/dL	39.42 ± 1.15	36.72 ± 0.93^∗^	35.90 ± 1.33^∗^	36.63 ± 3.24
Total WBC	/cmm	9, 200 ± 1, 593.74	6, 900 ± 1, 630.95	8, 680 ± 1, 227.60	7, 525 ± 1, 099.62
Eosinophil	%	0 ± 0	0 ± 0	0 ± 0	0 ± 0
Basophil	%	0 ± 0	0 ± 0	0 ± 0	0 ± 0
Band neutrophil	%	0 ± 0	0 ± 0	0 ± 0	0 ± 0
Segmented neutrophil	%	26.80 ± 7.98	20.00 ± 2.45	30.80 ± 20.44	27.75 ± 13.07
Lymphocytes	%	73.20 ± 7.98	80.00 ± 2.45	68.80 ± 21.25	72.55 ± 13.07
Monocytes	%	0 ± 0	0 ± 0	0 ± 0	0 ± 0
Platelet	/cmm	920, 200 ± 200, 671.87	1, 074, 000 ± 146, 145.48	1, 031, 600 ± 90, 262.40	898, 500 ± 267, 857.00

^∗^ *p* < 0.05, the significant difference compared to the control group using the one-way ANOVA continued by Tukey's multiple comparison test.

**Table 9:** Mean of the biochemical parameters of group rats which received polyherbal formulation or control before treatments in the subchronic oral toxicity study.

	Unit	Control	Dose		
			252 mg/kg	1,008 mg/kg	4,032 mg/kg
Male (*n* = 5)					
Urea	mg/dL	24.38 ± 5.54	30.14 ± 6.93	29.46 ± 8.71	26.32 ± 8.70
Creatinine	mg/dL	0.36 ± 0.05	0.38 ± 0.04	0.38 ± 0.04	0.40 ± 0.07
Total protein	mg/dL	5.60 ± 0.45	6.22 ± 0.79	5.96 ± 0.23	6.76 ± 0.30
Albumin	g/dL	3.46 ± 0.17	3.64 ± 0.28	3.48 ± 0.15	3.98 ± 0.31
Globulin	g/dL	2.14 ± 0.50	2.58 ± 0.85	2.48 ± 0.13	2.78 ± 0.45
AST (GOT)	U/L	198.72 ± 23.85	232.30 ± 12.11	230.08 ± 39.34	203.12 ± 16.11
ALT (GPT)	U/L	117.02 ± 20.33	139.30 ± 29.70	116.76 ± 17.59	109.66 ± 15.06

Female (*n* = 5)					
Urea	mg/dL	25.20 ± 8.39	28.16 ± 4.40	33.64 ± 2.23	35.00 ± 4.59
Creatinine	mg/dL	0.28 ± 0.04	0.36 ± 0.05	0.33 ± 0.04	0.36 ± 0.05
Total protein	mg/dL	5.68 ± 0.27	6.76 ± 0.25	6.14 ± 0.29	6.98 ± 0.88
Albumin	g/dL	3.50 ± 0.19	3.98 ± 0.19	3.72 ± 0.18	3.98 ± 0.38
Globulin	g/dL	2.18 ± 0.28	2.78 ± 0.19	2.42 ± 0.26	3.00 ± 0.57
AST (GOT)	U/L	193.34 ± 44.67	188.82 ± 31.48	191.54 ± 9.15	227.00 ± 9.77
ALT (GPT)	U/L	107.44 ± 22.06	113.02 ± 27.22	94.52 ± 6.49	111.74 ± 8.57

^∗^ *p* < 0.05, the significant difference compared to the control group using the one-way ANOVA continued by Tukey's multiple comparison test.

**Table 10:** Mean of the biochemical parameters of group rats which received polyherbal formulation or control after 45 days in the subchronic oral toxicity study.

	Unit	Control	Dose		
			252 mg/kg	1,008 mg/kg	4,032 mg/kg
Male (*n* = 5)					
Urea	mg/dL	45.25 ± 4.57	46.06 ± 4.34	37.60 ± 5.52	48.80 ± 4.30
Creatinine	mg/dL	0.46 ± 0.05	0.44 ± 0.05	0.40 ± 0.00	0.48 ± 0.04
Total protein	mg/dL	5.82 ± 0.49	5.02 ± 0.85	5.42 ± 0.36	7.66 ± 0.64^∗^
Albumin	g/dL	3.52 ± 0.23	3.86 ± 0.40	3.40 ± 0.37	3.62 ± 0.13
Globulin	g/dL	2.30 ± 0.50	1.16 ± 0.56^∗^	2.02 ± 0.08	4.04 ± 0.63^∗^
AST (GOT)	U/L	174.58 ± 27.79	225.70 ± 28.96^∗^	156.96 ± 24.41	190.78 ± 322.45
ALT (ALT)	U/L	110.48 ± 11.73	146.14 ± 37.14	91.52 ± 16.75	112.80 ± 27.45

Female (*n* = 5)					
Urea	Mg/dL	40.94 ± 9.04	47.36 ± 4.26	37.34 ± 5.34^∗^	48.30 ± 2.76
Creatinine	Mg/dL	0.54 ± 0.09	0.52 ± 0.13	0.42 ± 0.08	0.52 ± 0.04
Total protein	Mg/dL	6.32 ± 0.36	5.92 ± 0.43	5.64 ± 0.23	8.56 ± 0.67^∗^
Albumin	g/dL	4.04 ± 0.31	4.5 ± 0.42	3.86 ± 0.52	4.04 ± 0.23
Globulin	g/dL	2.32 ± 0.30	1.42 ± 0.50^∗^	1.72 ± 0.34	4.52 ± 0.57^∗^
AST (GOT)	U/L	148.92 ± 21.12	219.04 ± 63.70	196.70 ± 65.45	175.06 ± 39.34
ALT (ALT)	U/L	85.72 ± 16.17	144.30 ± 54.59	115.20 ± 42.03	100.30 ± 29.62

^∗^ *p* < 0.05, the significant difference compared to the control group using the one-way ANOVA continued by Tukey's multiple comparison test.

**Table 11:** Mean of the biochemical parameters of group rats which received polyherbal formulation or control after 91 days in the subchronic oral toxicity study.

	Unit	Control	Dose		
			252 mg/kg	1,008 mg/kg	4,032 mg/kg
Male (*n* = 5)					
Urea	mg/dL	30.92 ± 4.23	40.74 ± 8.12	31.80 ± 8.78	37.10 ± 2.33
Creatinine	mg/dL	0.46 ± 0.05	0.38 ± 0.08	0.48 ± 0.04	0.42 ± 0.04
Total protein	mg/dL	5.78 ± 0.47	6.14 ± 0.25	4.68 ± 0.87^∗^	5.32 ± 0.83
Albumin	g/dL	3.22 ± 0.16	4.06 ± 0.40	2.96 ± 0.58	3.50 ± 0.43
Globulin	g/dL	2.56 ± 0.42	2.08 ± 0.47	1.72 ± 0.56	2.62 ± 1.84
AST (GOT)	U/L	173.36 ± 25.80	249.36 ± 112.89	179.94 ± 27.40	219.44 ± 91.18
ALT (GPT)	U/L	145.82 ± 22.20	120.12 ± 30.49	112.06 ± 12.66	146.78 ± 37.48

Female (*n* = 5)					
Urea	mg/dL	37.02 ± 7.39	39.12 ± 7.96	37.64 ± 3.08	40.70 ± 6.09
Creatinine	mg/dL	0.40 ± 0.07	0.36 ± 0.09	0.54 ± 0.05	0.44 ± 0.05
Total protein	mg/dL	6.30 ± 0.16	6.72 ± 0.47	5.28 ± 0.78^∗^	5.44 ± 0.30
Albumin	g/dL	4.06 ± 0.32	4.20 ± 0.21	3.72 ± 0.29	3.74 ± 0.23
Globulin	g/dL	2.38 ± 0.26	2.52 ± 0.45	1.58 ± 0.48	1.70 ± 0.36
AST (GOT)	U/L	159.34 ± 16.66	166.82 ± 14.68	189.88 ± 42.24	153.82 ± 17.95
ALT (GPT)	U/L	110.08 ± 21.80	88.76 ± 25.08	106.00 ± 12.06	102.92 ± 5.78

^∗^ *p* < 0.05, the significant difference compared to the control group using the one-way ANOVA continued by Tukey's multiple comparison test.

**Table 12:** Mean of the absolute organ weight (g ± SD) of group rats which received polyherbal formulation or control after 91 days in the subchronic oral toxicity study.

Group and dose	Organ weight (g ± SD)									
	Heart	Liver	Lung	Stomach	Intestine	Kidney	Brain	Spleen	Testis	Ovary
Male										
I (252 mg/kg)	0.64 ± 0.04	5.28 ± 0.39	1.17 ± 0.19	1.81 ± 0.18	0.30 ± 0.09	0.63 ± 0.04	1.50 ± 0.18	0.43 ± 0.07	1.13 ± 0.34	
II (1,008 mg/kg)	0.77 ± 0.14	8.83 ± 1.06	1.21 ± 0.36	1.85 ± 0.29	0.27 ± 0.08	0.69 ± 0.07	1.69 ± 0.14	0.48 ± 0.13	1.31 ± 0.12	
III (4,032 mg/kg)	0.75 ± 0.18	8.40 ± 0.85	1.28 ± 0.13	1.95 ± 0.20	0.36 ± 0.09	0.72 ± 0.07	1.54 ± 0.20	0.53 ± 0.05	1.34 ± 0.12	
IV (water, control)	0.67 ± 0.06	7.99 ± 0.54	1.29 ± 0.24	1.86 ± 0.22	0.30 ± 0.07	0.72 ± 0.04	1.58 ± 0.12	0.51 ± 0.11	1.36 ± 0.19	

Female										
I (252 mg/kg)	0.60 ± 0.11	4.83 ± 0.37	1.17 ± 0.16	1.54 ± 0.17	0.29 ± 0.08	0.57 ± 0.06	1.46 ± 0.15	0.42 ± 0.06		0.59 ± 0.13
II (1,008 mg/kg)	0.60 ± 0.04	6.94 ± 0.86	1.21 ± 0.17	1.63 ± 0.15	0.33 ± 0.08	0.54 ± 0.10	1.54 ± 0.16	0.44 ± 0.05		0.70 ± 0.24
III (4,032 mg/kg)	0.65 ± 0.08	6.55 ± 0.79	1.14 ± 0.25	1.71 ± 0.16	0.34 ± 0.09	0.64 ± 0.08	1.60 ± 0.19	0.47 ± 0.06		0.66 ± 0.12
IV (water, control)	0.62 ± 0.04	6.43 ± 0.98	1.26 ± 0.28	1.77 ± 0.22	0.35 ± 0.06	0.62 ± 0.04	1.48 ± 0.12	0.51 ± 0.07		0.74 ± 0.17

^∗^ *p* < 0.05, the significant difference compared to the control group using the one-way ANOVA continued by Tukey's multiple comparison test.

**Table 13:** Mean of the relative organ weight (percentage of organ weight compared to body weight) of group rats which received polyherbal formulation or control after 91 days in the subchronic oral toxicity study.

Group and dose	Organ weight (%)									
	Heart	Liver	Lung	Stomach	Intestine	Kidney	Brain	Spleen	Testis	Ovary
Male										
I (252 mg/kg)	0.26 ± 0.04	2.16 ± 0.21	0.48 ± 0.06	0.75 ± 0.10	0.12 ± 0.03	0.26 ± 0.03	0.62 ± 0.09	0.18 ± 0.02	0.46 ± 0.15	
II (1,008 mg/kg)	0.32 ± 0.06	3.63 ± 0.53	0.49 ± 0.14	0.76 ± 0.13	0.11 ± 0.04	0.29 ± 0.02	0.69 ± 0.07	0.16 ± 0.02	0.54 ± 0.04	
III (4,032 mg/kg)	0.31 ± 0.09	3.46 ± 0.27	0.53 ± 0.07	0.80 ± 0.11	0.15 ± 0.04	0.31 ± 0.03	0.63 ± 0.07	0.22 ± 0.03	0.55 ± 0.06	
IV (water, control)	0.27 ± 0.02	3.20 ± 0.19	0.52 ± 0.09	0.75 ± 0.11	0.12 ± 0.03	0.29 ± 0.02	0.63 ± 0.07	0.21 ± 0.05	0.55 ± 0.08	

Female										
I (252 mg/kg)	0.30 ± 0.06	2.42 ± 0.24	0.58 ± 0.07	0.77 ± 0.09	0.14 ± 0.03	0.29 ± 0.04	0.73 ± 0.10	0.21 ± 0.04		0.29 ± 0.06
II (1,008 mg/kg)	0.30 ± 0.02	3.47 ± 0.40	0.61 ± 0.07	0.82 ± 0.07	0.17 ± 0.04	0.28 ± 0.02	0.78 ± 0.09	0.22 ± 0.03		0.35 ± 0.13
III (4,032 mg/kg)	0.34 ± 0.04	3.42 ± 0.42	0.59 ± 0.10	0.89 ± 0.10	0.18 ± 0.05	0.35 ± 0.05	0.84 ± 0.11	0.24 ± 0.03		0.35 ± 0.06
IV (water, control)	0.33 ± 0.03	3.47 ± 0.64	0.68 ± 0.15	0.71 ± 0.06	0.19 ± 0.04	0.33 ± 0.04	0.79 ± 0.07	0.28 ± 0.04		0.40 ± 0.09

^∗^ *p* < 0.05, the significant difference compared to the control group using the one-way ANOVA continued by Tukey's multiple comparison test.

## Data Availability

The data used to support the findings of this study are available from the corresponding author upon request.
